# Tracheostomy in infants with severe bronchopulmonary dysplasia: A review

**DOI:** 10.3389/fped.2022.1066367

**Published:** 2023-01-12

**Authors:** Gangaram Akangire, Winston Manimtim

**Affiliations:** ^1^ Division of Neonatology, Children's Mercy Kansas City, Kansas City, MO, United States; ^2^ Department of Pediatrics, School of Medicine, University of Missouri-Kansas City, Kansas City, MO, United States

**Keywords:** tracheostomy (TS), bronchopulmonary dysplasia, reshospitalization, home ventilation, airway disorders, chronic lung disease (CLD) of prematurity, neonatal outcomes

## Abstract

In recent years, with increased survival of infants with severe bronchopulmonary dysplasia (BPD), long term ventilation due to severe BPD has increased and become the most common indication for tracheostomy in infants less than one year of age. Evidence shows that tracheostomy in severe BPD may improve short- and long-term respiratory and neurodevelopmental outcomes. However, there is significant variation among centers in the indication, timing, intensive care management, and follow-up care after hospital discharge of infants with severe BPD who received tracheostomy for chronic ventilation. The timing of liberation from the ventilator, odds of decannulation, rate of rehospitalization, growth, and neurodevelopment are all clinically important outcomes that can guide both clinicians and parents to make a well-informed decision when choosing tracheostomy and long-term assisted ventilation for infants with severe BPD. This review summarizes the current literature regarding the indications and timing of tracheostomy placement in infants with severe BPD, highlights center variability in both intensive care and outpatient follow-up settings, and describes outcomes of infants with severe BPD who received tracheostomy.

## 1. Introduction

Pediatric tracheostomy has increased significantly in recent years, particularly in children less than one year of age (1–4). Prolonged mechanical ventilation in post-prematurity infants with severe bronchopulmonary dysplasia (BPD) is now the most common indication for tracheostomy in infants (5, 6). As advanced technology in neonatal intensive care units (NICUs) becomes more routine, the survival of extremely premature infants has also increased the need for long-term mechanical ventilation (7), specifically for those infants born at the threshold of viability (≤24 weeks' gestation), who have greater odds of developing severe BPD (8, 9). Other reasons for performing tracheostomy in infants include congenital anomalies of the airway, neurological and complex cardiac anomalies, and genetic diseases with either short- or long-term survival outcome (7, 8, 10).

A recent study found that the rate of tracheostomy in infants increased from 1.9 to 3.5 per 100,000 live births between 2011 and 2017 with a corresponding increase in hospital costs (8). Murthy et al. reported that among members of the Children's Hospital Neonatal Consortium (CHNC), the incidence of tracheostomy varied between centers ranging from as low as 2% to as high as 37%, showing that the indication of tracheostomy in severe BPD infants remains uncertain (11). The timing or age when tracheostomy was performed also varied across institutions, as generally accepted guidelines do not exist. Although various authors have described management of ventilator-dependent infants with severe BPD in intensive care settings (12–15), a recent multicenter collaborative study found significant variation in the mode of respiratory support provided to these infants (16). In an effort to standardize the care of ventilator-dependent infants and children through a tracheostomy after hospital discharge, the American Thoracic Society (ATS) has published clinical practice guidelines (17). However, significant institutional variation exists in follow-up care models and discharge destinations (18, 19). The literature on post-discharge outcomes of infants with tracheostomy is very limited. Outcomes that are ultimately important in this vulnerable population include the survival rate, liberation from the ventilator, rate of tracheostomy decannulation, growth, and neurodevelopmental trajectories.

Our goal in this review is to provide a synopsis of currently available studies that have reported the indications, rate, and timing of tracheostomy in infants with severe BPD, including the available post-hospital discharge survival, respiratory, growth and neurodevelopmental outcomes. The available knowledge about these outcomes, while limited, will help clinicians navigate the difficulty of counseling families and caregivers who may choose the path of tracheostomy and long-term assisted ventilation of infants with severe BPD.

### 1.1. Indications for tracheostomy in infants with severe BPD

In the last few decades, the most common indications for tracheostomy in children have changed from acute infections, such as epiglottitis and croup, to prolonged mechanical ventilation in infants (20). BPD, or chronic lung disease (CLD), comprises almost 50% of infants needing tracheostomy, followed by congenital or acquired airway abnormalities (vocal cord paralysis, tracheobronchomalacia (TBM), subglottic stenosis), craniofacial anomalies, and cardiac, neurologic or musculoskeletal, and genetic disorders (19, 21, 22). Recently, in addition to BPD or CLD, rare genetic anomalies in infants are increasing indications for receiving tracheostomy to sustain survival outside of intensive care settings (1, 23, 24).

Specific to infants with severe BPD, tracheostomy is performed based on several factors including center-specific practices, corrected age and clinical status of the infant, and parental preferences (5, 19). The decision to offer tracheostomy may also depend on the availability of a home ventilator follow-up program within the hospital's geographical location. Traditionally, tracheostomy is considered for infants who are unable to be weaned off from mechanical ventilation. To facilitate successful extubation, prevent reintubation, and minimize the consequences of prolonged invasive ventilation, various modes of non-invasive ventilatory (NIV) support have been used, that include non-invasive neurally adjusted ventilatory assist (NIV-NAVA), non-invasive positive pressure ventilation (NIPPV), nasal continuous positive airway pressure (CPAP) and high flow nasal cannula (HFNC). A recent study suggests that extubation to NIV-NAVA may be a more successful strategy than to either nasal CPAP or NIPPV (25, 26). The reasons for failure of extubation to NIV may include patient's asynchrony with the ventilator, or patient's inability to trigger the ventilator, or use of ineffective nasal interphase. Tracheostomy is also indicated in infants with severe BPD who have difficulty in achieving adequate growth and developmental milestones with advancing gestational age. However, high cost of tracheostomy and subsequent follow-up may limit its use in infants who require prolonged respiratory support.

Airway complications from prolonged intubation, such as subglottic stenosis or TBM that require tracheostomy, have been observed more frequently in infants with severe BPD (27). In infants with severe BPD and TBM, a continuous level of positive end-expiratory pressure (PEEP) directly applied into the airway may be needed to stent the airway open during exhalation, as described in a recent case report of severe TBM in a ventilator-dependent infant with BPD who required a combination of NAVA ventilation and a PEEP of up to 20 cm H2O during episodes of severe airway obstruction (28).

The development of BPD-associated pulmonary hypertension has been found to be independently associated with the need for tracheostomy in infants with severe BPD (24). Poor growth and delayed development may also contribute to the need for long-term mechanical ventilation through a tracheostomy in infants with severe BPD. A recent survey among 29 responders from 34 tertiary care centers participating in the Children's Hospitals Neonatal Consortium (CHNC) reported that, the most common criteria that contributed to the decision for tracheostomy in infants with severe BPD were airway and ventilation (32%), pulmonary hypertension (16%), multiple courses of corticosteroid therapy (11%), and failure to thrive on noninvasive support (11%) (5). In this same study, the ranges of clinical parameters that would prompt discussion of tracheostomy included pCO2 76–85 mmHg, FiO2 > 0.60, PEEP 9 cm–11 cm H2O, respiratory rate 61–70 breaths/min consistently, post menstrual age (PMA) > 44 weeks, and weight <10th percentile at 44 weeks PMA (5).

In our institution, the decision to place a tracheostomy in infants with severe BPD for purposes of long-term ventilation at home is arrived at after a multidisciplinary team discussion and parental acceptance. A gastrostomy tube is usually placed at the same time in anticipation that these infants will have limited ability to take feedings by mouth.

### 1.2. Timing of tracheostomy in infants with severe BPD

Currently, no consensus recommendation exists for the optimal timing of placement of tracheostomy in infants with severe BPD. Most single-center studies have reported the timing of tracheostomy as ranging between 42 and 51 weeks PMA (7, 19, 29–31). This variation in timing may be related to multiple factors but presumably indicates individual institutional practices, clinician, or parental preferences. It is also unclear whether the timing of tracheostomy has any impact on long-term respiratory, growth, and neurodevelopmental outcomes. It was reported that after placement of a tracheostomy in infants with severe BPD while in the NICU, sustained adequate growth and active participation in neurodevelopmental activities was observed, leading to improved outcomes (32). In a large multicenter study, Demauro et al. reported that the odds of death or neurodevelopmental impairment at 18–22 months of age are lower in infants who received tracheostomies before, rather than after, 120 days of life (aOR 0.5, 95% CI 0.3–0.9) (1).

### 1.3. Post-tracheostomy management of infants with severe BPD

Immediately following tracheostomy placements, these infants are expected to be either medically paralyzed or heavily sedated in the first few days to a week until the first tracheostomy tube change is accomplished based on local institutional practices (12). Once paralysis and/or sedation medications are weaned, the ventilator settings are adjusted based on the patient's clinical parameters. Frequently, these infants may require higher ventilator settings post-tracheostomy compared to pre-tracheostomy level of respiratory support. A previous study in our institution observed that infants with evidence of pulmonary hypertension on echocardiogram were at higher risk for clinical deterioration post tracheostomy, likely related to resurgence of pulmonary hypertensive crisis (33). Respiratory infections that develop soon after tracheostomy placement may also cause respiratory deterioration in some infants (12). Often, uncontrolled pain and ongoing need for pain medications and/or sedation prevent stabilization of these infants postoperatively (12). Variation exists across institutions on the choice of cuffed or uncuffed tracheostomy tubes. In general, if tracheostomy is performed for an upper airway obstruction and if patient is not ventilator dependent, uncuffed tracheostomy tube is preferred. Patients with severe BPD are most likely ventilator dependent and some institutions including ours prefer cuffed tube to optimize the delivery of high pressures and tidal volume by allowing only minimal leak. Minimal leak point is achieved by adding water to the cuff while auscultating the airway at laryngeal level until leak is not heard during inspiration, then, some water is withdrawn until a small leak is heard. Some ventilators have the ability to provide % leak which can be utilized as well to determine minimal leak. Although gestational age is a driving factor to choose the initial tracheostomy size and length, anatomical differences play a significant role. Upsizing the tracheostomy is determined by otolaryngologists based on the bedside flexible endoscopy through tracheostomy. During this procedure it is ensured that there is enough space above carina so a longer tracheostomy would fit well. If there is significant tracheomalacia, a longer tracheostomy is preferred to bypass the proximal tracheomalacia. Large tracheostomy tubes have less chance of plugging and accidental decannulation but decrease phonation when child tries to vocalize. Otolaryngologists perform a bronchoscopy after tracheostomy and prior to hospital discharge, and the tracheostomy tube is upsized then if needed based on the findings. Tracheostomy granulomas, stoma breakdown, stoma infection, and tracheostomy tube plugging are some of the common complications. Tracheostomy granulomas are typically managed by using topical steroid and antibiotic combination cream for several days. Stoma break down is typically caused by tracheostomy tube pressing or rubbing on the skin and can be prevented by using dressings like simple gauze, tritec silver or tritec ultra for 1–2 weeks after tracheostomy placement. Stoma infection is treated with topical antibiotic cream and in some instances, systemic antibiotic is needed. Tracheostomy tube plugging can be prevented by maintaining adequate hydration status of the patient and providing adequate moisture in respiratory circuit and airway.

McKinney, et al. recently reported that, among 15 BPD Collaborative academic centers, there was a significant variation in mode and settings among centers for both invasive and noninvasive ventilation for infants with established severe BPD (16). Among those receiving invasive ventilation, 53% had tracheostomy, and synchronized intermittent mandatory ventilation (SIMV) was most frequently utilized, with volume control (VC) or volume guaranteed (VG) mode more commonly used than pressure control (PC) mode. Only a small number (6%) used neurally adjusted ventilatory assist (NAVA) mode (34). A recent multicenter experience with NAVA in infants with severe BPD concluded that NAVA can be used safely and effectively in select infants with severe BPD (16). In addition, NAVA when used as a sequel mode of ventilation in infants with evolving or established BPD showed similar respiratory outcomes compared to conventional ventilation and may decrease the need for sedation (35). Nevertheless, the variation in ventilator modes and settings among centers is likely multifactorial in nature and highlights the need for a prospective trial to determine what ventilation strategies are most effective for infants with established severe BPD based on the specific BPD phenotype.

### 1.4. Transitioning from ICU ventilator to home ventilator

The current literature is very limited on how infants with severe BPD who received tracheostomy are transitioned to portable ventilators that can be used outside of intensive care settings. Because there are technological limitations to the degree and mode of respiratory support that portable ventilators can provide at home, ventilatory support after tracheostomy placement in infants with severe BPD must be weaned to a level that can be accommodated by a portable ventilator.

Once a period of clinical stability is achieved, ventilator settings are adjusted with the goal of weaning settings to levels that can be matched on the portable home ventilator. This desired level may take several weeks to months to reach. There are several types of home ventilators available in the market that have their own specific capabilities, unique features, or single vs. double limb circuits. Most ventilators designed for home use may have a minimum weight requirement of at least 5 kg (12). Each institution may have its own preference which home ventilator to prescribe, and it depends most likely on the medical provider's own experience. Some institutions are left with the option of using whatever home ventilator is supplied by the local durable medical equipment (DME) company. There has not been any study that compared the effectiveness of the different types of home ventilators for ventilator-dependent infants with BPD. However, a quality improvement study published recently reported that using a standard protocol of transitioning from an ICU to a portable ventilator increased the success rate and earlier transition to home ventilator (36). During transition, tidal volume on the portable ventilator is increased by 5–10 ml, PEEP and peak inspiratory pressure (PIP) may be increased based on patients' tolerance to change, work of breathing, and FiO2 change. Trigger sensitivity of portable ventilator is adjusted based on patients' needs, synchrony with triggering and type of portable ventilator provided by local DME company. Depending on the type of portable home ventilator, triggering sensitivity level can range from 0.25 L/minute to 1 L/minute and may have to be adjusted based on patients' ability to trigger ventilator supported breaths consistently. The success of transition to a home ventilator depends on multiple factors such as the severity and specific phenotype of the BPD disease, presence of co-morbidities, growth velocity and the required level of respiratory support. More generalizable clinical practice guidelines are urgently needed that can be adopted by most centers caring for infants with BPD who received tracheostomy and are being transitioned to home ventilators.

### 1.5. Discharge criteria, preparation, and destination

In 2016, the American Thoracic Society (ATS) published an official clinical practice guideline for pediatric chronic home invasive ventilation (17). Based on available evidence, this document detailed the preparation, education, and skills training for at least two caregivers in the home to be able to care safely for a child on a ventilator at home. Further, the document described the requirements for a caregiver who is always awake, the use of monitoring devices such as pulse oximeter, and the provision of all equipment by a DME company. The document also emphasized the importance of securing skilled nursing for support at home (17).

In our institution, parents and/or family caregivers of an infant with tracheostomy are required to take multiple classes as part of educational training (19) on cardiopulmonary resuscitation, which includes simulation sessions using different emergency scenarios that could happen at home. Primary caregivers are required to demonstrate competency in various skills, such as tracheostomy tube changes, tracheostomy care, airway suctioning, and respiratory medication administration. Parents are asked to conduct a stroller ride to familiarize themselves with packing supplies and portable equipment to use during travel. Following these classes, parents are asked to stay in a parent care unit where they care for their infants on their own for 48 h continuously to simulate the real home environment. Typically, private duty nursing coverage is required during the first two weeks of being home. However, due to the recent national shortage of availability of skilled home nurses (37), parents are encouraged to rely on additional caregivers including family, relatives, and friends to provide respite care. A published quality improvement project reported that using a standardized discharge process that included educational materials, a chronic ventilation road map for caregivers, electronic tracking of discharge readiness, team-based care coordination, and timely arrangement for home nursing significantly decreased the length of hospital stay and cut the cost of hospitalization without compromising the safety of ventilator-dependent children with tracheostomy discharged to home (38).

The discharge destination for children receiving invasive ventilation through a tracheostomy may vary depending on the institution's local practice model and the available resources in the geographic area where care is provided. In some states, long-term care facilities that can provide chronic respiratory and rehabilitative services are available to which infants with tracheostomy and ventilator dependence can be discharged as a bridge to going home eventually. This type of accommodation will allow parents and family caregivers to continue to learn the skills necessary to care for their technology-dependent children in a setting with much less intensive care. Although this type of arrangement facilitates earlier discharge from the ICU, it is not readily available in most cities and, where it is, the waiting period for the next available space is usually quite long. As an alternative, some children's hospitals have specialized transition or step-down units for patients with tracheostomy where they can be cared for until certain respiratory support status and set criteria are met for discharge to home. Foster care parenting is sometimes pursued legally for some of these medically complex children whose parents may have significant psychosocial issues. This situation occurs in our institution at a rate of 9%–10% among our ventilator-dependent infants with tracheostomy (19).

### 1.6. Follow-up care and home management of infants with severe BPD who received tracheostomy

According to the ATS evidence-based clinical practice guidelines for hospital discharge and management of children requiring chronic invasive ventilation at home and in the community (17), a collaborative generalist and subspecialist co-management medical home model is most likely to be successful for the care of children requiring chronic home invasive ventilation. In addition, several authors have described their center's follow-up care models after hospital discharge of this cohort of chronically ventilated children (7, 31). In our institution, the coordinated and complex multidisciplinary medical care provided to ventilator-dependent infants with severe BPD and discharged to home is unique in that it is led by the same group of dedicated neonatologists who cared for them while they were in the NICU. Additionally, these neonatologists also provide both primary and subspecialty care in the medical home clinic supported by a dedicated pulmonologist, otolaryngologists, gastroenterologist, the pulmonary hypertension team, and allied medical health providers (19). As new technologies are developed for home use, as the number of technology-dependent infants discharged from intensive care settings continues to grow, and as evidence of their outcomes becomes available, medical providers, parents, and families, and the community-at-large will be better informed about the comprehensive care needed to manage this unique subpopulation of high-risk and medically complex children in the comfort of their home.

### 1.7. Ventilator weaning, decannulation, growth, and neurodevelopmental outcomes of infants with severe BPD who received tracheostomy

#### 1.7.1. Weaning from the home ventilator and tracheostomy decannulation

To date, there are no universal guidelines for weaning from the ventilator for those children who are chronically ventilated at home through a tracheostomy. Furthermore, institutions vary in the frequency of follow-up clinic visits that may preclude early and aggressive weaning of these patients from the ventilator. Some centers have incorporated telehealth visits during the COVID-19 pandemic to wean their clinically stable patients virtually from the ventilator. Nevertheless, an increasing number of single-center studies have been published describing individual centers' own method of weaning from the home ventilator and their outcomes. The first comprehensive study by Cristea et al., in 2013, reported a cohort of 102 patients with severe BPD and tracheostomy and showed that, of the infants who survived during the study period, 83% were liberated from the home ventilator, of which 97% were liberated from home ventilator by five years of age, with a median age of liberation from the ventilator of 24 months (31). Following liberation from the ventilator, a median period of 11 months elapsed between ventilator liberation and tracheostomy decannulation (31). More recent studies, including one from our center, have shown a similar median age of liberation from the ventilator and age of decannulation (7, 19, 39–41). Specific to our cohort, we found that ventilator-dependent infants with severe BPD have much greater odds of successful weaning from the ventilator and decannulation by three to four years of age, compared to those infants with other indications for tracheostomy and chronic invasive ventilation at home (19, 42). Table 1 summarizes the respiratory outcomes to date of infants with severe BPD who were chronically ventilated at home through a tracheostomy. There are also center variations in terms of methods for weaning infants from the ventilator. Examples may include daytime first followed by nighttime weaning, performance of polysomnogram during nocturnal weaning, transition to CPAP, or overnight hospital admission to wean from the ventilator (39, 43–45).

**Table 1:** Summary of respiratory outcomes of infants with severe BPD who received chronic ventilation at home through a tracheostomy.

Authors, year of publication	Patient Population	Primary Indication for Tracheostomy	Age at Tracheostomy	Rate (%) and Age at liberation from home ventilator	Rate (%) and Age at decannulation	Survival rate (%) after hospital discharge	Follow-up period
Cristea et al., 2013	Preterm infants born at 26 weeks ** (IQR 25, 27) (*n* = 102)	Severe BPD	Not reported	97.1% 24 months**(IQR-19–33)	58.8% 37.5 months** (IQR-31.5–45)	81.4%	Up to 5–6 years of age (total of 871 person years)
Akangire et al., 2020	Ventilator-dependent infants (n = 204) BPD only (*n* = 82)^#^	Severe BPD Airway Cardiac Neurologic Genetic	4.9 months* (SD 4.6)	100% 27.2 months*(SD 23)	50% 41.9 months*(SD 23.3)	80.5%	Up to 4 years of age
House et al., 2021	Preterm infants born at < 33 weeks (*n* = 49)	Severe BPD	43.3 weeks* (range 36.3-56.9)	97.0% 27.3 months* (SD 4.1, range 7–45 months)	79% 44.7 months* (range- 22–79)	73.9%	Up to 5 years of age
Sillers et al., 2021	Tracheostomy-dependent infants (*n* = 323) CLD/BPD only (*n* = 180)^#^	Pulmonary Anatomic Cardiac Neurologic Musculoskeletal Other	52.3 weeks PMA** (IQR 44.3, 60.0)	Not reported	51.3% 3.1 years** (IQR 2.4, 4.5)	82%	Minimum of 3 years after tracheostomy placement
Akangire et al., 2022	Tracheostomy dependent infants (*n* = 98)	Severe BPD	43 weeks PMA or 4 months** (IQR 3, 5)	100%, 24 months** (IQR 18, 29)	52%, 32 months** (IQR 26, 39)	99%	Up to 4 years of age

*Mean.

** Median.

^#^ Outcomes for CLD/BPD subgroup only.

Once these children are weaned off the ventilator and continue to improve and thrive, the introduction and demonstration of tolerance for using a speaking valve (Passy Muir) to facilitate swallowing, vocalization, and improved secretion clearance (46, 47), followed by tracheostomy tube capping during awake periods, may be prescribed. In our institution, as in many other institutions, to prepare for decannulation, we perform a routine surveillance airway bronchoscopy to look for possible airway obstructive lesions or dynamic airway collapse and a capped overnight sleep study in the sleep lab (39, 43, 45, 48–51), to ensure successful and safe decannulation. Post decannulation, otolaryngologists and pediatric pulmonologists follow these patients for the management of tracheostomy stoma, other airway complications, and long-term respiratory morbidities related to severe BPD (19).

#### 1.7.2. Growth outcomes

Several studies have indicated that nutrition is a key component of lung growth, particularly for infants with severe BPD who require tracheostomy and home ventilation (52–55). These infants have high energy needs and energy consumption, manifested by increased work of breathing in an effort to mitigate ongoing lung inflammation and sustain continuous lung repair (56). The study by Luo et al. found that most of these infants were born AGA and yet were severely and disproportionately growth restricted by the time of tracheostomy suggesting that these infants suffer from postnatal growth failure in the early weeks of NICU hospitalization. The median weight z-score decreased from −0.45 at birth to −1.42, with >41% of infants had z-score <2 at the time of tracheostomy. The length *z*-score also decreased from −0.64 at birth to −3.07 with >63% of patients having *z*-score <2 at the time of tracheostomy (32). Luo also found that these infants had significant improvement in their z-scores for weight, length, weight/length ratio despite decreased caloric intake after receiving tracheostomy while in the NICU (32). On the other hand, infants who were born already small for gestational age (SGA) continue to suffer from postnatal growth failure while in the NICU and were found to be at much greater risk for worse outcomes (57). These findings underscore the role of a dedicated dietician to optimize nutritional and feeding support for all infants with severe BPD prior to and after tracheostomy while in the NICU.

There is limited literature on the post-hospitalization growth outcomes of infants with severe BPD who received tracheostomy. Typically, these infants are followed by pediatricians who work collaboratively with dietitians. Most of these infants are discharged on gastrostomy tube feeding to optimize delivery of nutrition (30, 57, 58). The unique aspect of our medical home care model for infants with severe BPD and tracheostomy renders us the ability to optimize their nutritional support and manage any coexisting feeding problems, which are very common in this patient population (19). We reported previously that ventilator-dependent infants with tracheostomy had improved z-scores for weight and weight for length at hospital discharge that remained consistent through three years of age, a testament to the dedicated dietitian's role that extends into the outpatient follow-up clinic (19). One area of research that must be explored is whether providing optimal nutrition to improve growth has an impact on weaning from ventilator support that leads to successful decannulation in infants with severe BPD who received tracheostomy.

#### 1.7.3. Neurodevelopmental outcomes

Infants with severe BPD who received tracheostomy were found to have improved short-term outcomes while in the NICU. These infants were able to tolerate active participation in various developmental activities without respiratory compromise (32). In addition, they were also found to have less need for sedation medications with their potential negative effect on neurodevelopment (32). In a large multicenter study that included 304 preterm infants who received tracheostomy, Demauro et al. found that the odds of death or neurodevelopmental impairment are high (OR 3.3, 95% CI 2.4–4.6) and that tracheostomy alone cannot mitigate the significant risk associated with many complications of prematurity (1). Furthermore, Annesi et al. reported that infants with severe BPD and tracheostomy suffered from increased long-term cognitive delay beyond 24 months of age (59). On the other hand, Cammack et al. observed no difference in cognitive and language development at two years of age in infants with severe BPD who received tracheostomy compared to those without a tracheostomy (60). As more and more extremely preterm infants with severe BPD are surviving to hospital discharge with a tracheostomy for chronic invasive ventilation, the need for multicenter research to determine their long-term neurodevelopmental outcomes and identify modifiable risk factors for worst outcomes become imperative.

### 1.8. Mortality and rehospitalizations

The mortality rate after hospital discharge of infants with severe BPD who received tracheostomy ranged between 15% and 21% as reported by several authors (19, 22, 31). (Table 1) Cristea et al. reported no significant difference in the demographic data in their cohort between those who died and those who survived (31). Akangire et al. reported that of the 21% of their patients who died by four years of age, the median age of death occurred at 27 months (19), while Sillers et al. found a median age of death to be 17 months (22). The most common cause of death after discharge from the hospital was cardiopulmonary arrest due to accidental tracheostomy decannulation or plugging (7, 19). Interestingly, Cristea et al., in a study of 94 infants with severe BPD and tracheostomy found significantly higher mortality among economically disadvantaged families with income below the state median household income, or those who reside in poor geographic Zip codes (61).

On the other hand, the overall mortality rate including those who died after tracheostomy but before hospital discharge was about 26% (7, 22). The cause of death for these patients varied depending on the primary indication for tracheostomy (22), or prematurity-related issues, redirection of care due to poor neurologic prognosis, or respiratory failure related to severe pulmonary hypertensive crisis (7). Additionally, being born SGA was found to be a significant risk factor for death. However, the degree of respiratory support as measured by mean airway pressure and fraction of inspired oxygen at the time of tracheostomy were not found to be associated with the risk for death (7).

Information about the rehospitalization of ventilator-dependent infants with severe BPD with a tracheostomy is very limited. Several reasons may be related to the nature and geographical location of where these infants receive both their primary and subspecialty care, i.e., community-based care setting vs. academic or tertiary hospital-based clinics with availability of pediatric subspecialty support including pediatric pulmonology and otolaryngology. Akangire et al. reported respiratory viral infections specifically caused by rhino-enterovirus as the most common cause of rehospitalization. Other causes include non-infectious respiratory conditions, equipment malfunction, feeding or gastroenterology-related causes, and need for surgical procedures. In the same study, infants who were ventilator and oxygen-dependent, and on chronic use of inhaled corticosteroid were significantly at higher risk of rehospitalization in the first two years of life (62).
